# R583Q CACNA1A variant in SHM1 and ataxia: case report and literature update

**DOI:** 10.1007/s10194-012-0444-7

**Published:** 2012-04-19

**Authors:** Andrea Di Cristofori, Laura Fusi, Antonella Gomitoni, Giampiero Grampa, Anna Bersano

**Affiliations:** 1 Department of Neurosurgery, University of Milan, Milan, Italy; 2 IRCCS Foundation Ospedale Maggiore Policlinico Mangiagalli and Regina Elena, Via F. Sforza 35, 20122 Milan, Italy; 3 Department of Neurological Sciences, IRCCS Foundation Ospedale Maggiore Policlinico Mangiagalli and Regina Elena, Via F. Sforza 35, 20122 Milan, Italy; 4 Stroke Unit, Azienda Ospedaliera di Circolo di Busto Arsizio, Saronno, Tradate, Saronno Italy; 5 Department of Neurological Sciences, University of Milan, Milan, Italy; 6 Department of Emergency Neurology, IRCCS Foundation Neurological Institute C.Mondino, Pavia, Via Mondino 2, 27100 Pavia, Italy

**Keywords:** FHM, SHM, Ataxia, Cerebellar, CACNA1A

## Abstract

Familial hemiplegic migraine (FHM) type 1 is a rare monogenic dominant autosomal disease due to *CACNA1A* gene mutations. Besides the classical phenotype, mutations on *CACNA1A* gene are associated with a broader spectrum of clinical features including cerebellar ataxia, making FHM1 a complex channelopathy. We report the case of a patient carrying the p.Arg583Gln mutation affected by hemiplegic migraine and late onset ataxia and we performed a literature review about the clinical features of p.Arg583Gln. Although p.Arg583Gln mutations are associated with a heterogeneous phenotype, carriers present cerebellar signs which consisted generally in ataxia and dysmetria, with intention tremor appearing mostly in advanced age, often progressive and permanent. The heterogeneous spectrum of *CACNA1A* gene mutations probably causes sporadic hemiplegic migraine (SHM) to be misdiagnosed. Given the therapeutic opportunities, SHM/FHM1 should be considered in differential diagnosis of patients with cerebellar ataxia and migraine with aura.

## Introduction

Familial/sporadic hemiplegic migraine (FHM/SHM) is a rare migraine with aura subtype, usually inherited in an autosomal dominant manner [1]. Mutations on *CACNA1A* on chromosome 19p13, (FHM1), *ATP1A2* on chromosome 1q23 (FHM2) and *SCN1A* genes on chromosome 2q24 (FHM3) were identified as causing FHM [2, 3]. Missense mutations in the *CACNA1A* gene, which encodes for the alpha1 subunit of a P/Q type voltage-gated calcium channel, account for 50–70 % of FHM patients [2]. Besides hemiplegic migraine, FHM1 is associated with a broad spectrum of clinical features [3, 4]. Cerebellar signs and symptoms, ranging from nystagmus to progressive, usually late-onset, mild ataxia, both during or independently from FHM/SHM attacks, are also not uncommon in FHM1 families [3–9]. They have been identified in up to 20–40 % of FHM1 families and can become permanent and associated with cerebellar atrophy at cerebral MRI in 60 % of affected individuals. Pathogenesis of cerebellar involvement remains controversial. It has been shown that different FHM mutations induce changes in single-channel function and expression leading to opposite effects on Ca^2+^ influx. Particularly, some authors observed that only mutations causing a reduction of Ca^2+^ influx are associated with cerebellar ataxia in addition to the classical FHM phenotype, whereas in other papers cerebellar signs have been associated with a gain of function in Ca^2+^ receptor determining lower threshold and lower depolarization level [4, 10]. We report a 54-year-old woman, among the population of the Lombardia GENS project (http://www.clinicaltrial.gov), affected by migraine with aura and ataxia carrying the p.Arg583Gln missense substitution in the *CACNA1A* gene focusing, through a review literature, on the clinical phenotype of mutation carriers.

## Case report

**Fig. 1:**
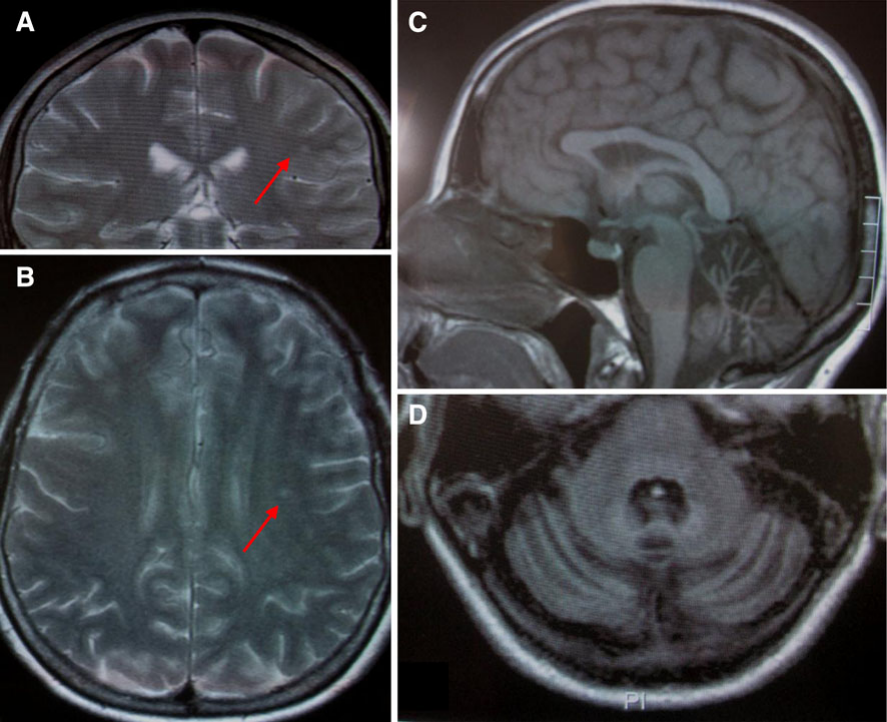
**a**, **b** Coronal and axial T2-weighted images showing the small vascular lesion near the left silvian scissure (*red arrow*). **c**, **d** Axial and sagittal T1 weighted images demonstrating cerebellar atrophy with deeper sulci on (color figure online)

A 54-year-old woman (PV) was admitted in 2009 to the Stroke Unit of Azienda Ospedaliera Ospedale di Circolo di Busto Arsizio, Saronno, Tradate for recurrent attacks of right temporal migraine followed by a sudden sequence of left hemiparesis, associated with a decreased level of consciousness, dysarthria, vision loss and sensory deficit alternating in limb side. The neurological examination at admission revealed a mild depression and a slight cognitive deficit with a Minimental State Examination Score (MMSE) of 27/30. No cranial nerve deficits, including dysarthria and nystagmus, were detected. A slight left hemiparesis, mild hypotonia in the four limbs and alternating arm deficit in sensation were also observed. Cerebellar tasks showed a bilateral dysmetria pattern at the nose–finger and heel–shin test and inaccuracies were observed in fast alternating hand movements (dysdiadochokinesia). Truncal ataxia and intention tremor were not detected. Stance was achievable without aid although the loss of balance and gait was ataxic, but she was able to walk without help. From early childhood she suffered from migraine with aura with a bi-monthly frequency, mainly characterised by vision loss, sensory deficit in the right limbs and sometimes, left hemiparesis. Aura usually developed over a period of 5 min and lasted less than 60 min. No triggers for symptoms onset or worsening were identified. Biochemistry, inflammatory markers, thyroid hormones, cyanocobalamin dosage and autoantibodies search (anti GAD, anti-glyadin, anti-endomysium, anti neuronal) were negative. ECG, echocardiography and epiaortic and transcranial ultrasound examination were normal too. The brain CT scan performed in the acute phase demonstrated a hypodense round lesion proximal to the left silvian fissure. The cerebral MRI confirmed the left hyperintense round lesion at T2 and FLAIR weighted images, consistent with an old small ischemic infarction and revealed a mild cerebellar atrophy (Fig. 1). Electroencephalogram showed unspecific abnormalities on the left hemisphere, consistent with lesional activity. During the hospitalization, the patient’s left strength deficit as well as migraine progressively improved whereas cerebellar deficits remained stable. Since the patient did not reported other hemiplegic attacks at 1 year follow-up, no therapy was administered. Familial history was not completely available given the premature death of both parents. One brother had an undocumented mental retardation. However, his neurological examination was negative except for a bradypsychia.

Given the suspicion of sporadic hemiplegic migraine, a genetic analysis for FHM1 and FHM2 was performed after obtaining informed consent. Sequence analysis of *CACNA1A* gene revealed the presence of the heterozygous variant c.1748G>A, resulting in the p.Arg583Gln missense substitution in the putative protein, already described in association with hemiplegic migraine and ataxia (http://grenada.lumc.nl/LOVD2/FHM/home.php).

## Discussion

We report a patient presenting with migraine with aura and cerebellar signs and symptoms, carrying the p.Arg583Gln mutation in the *CACNA1A* gene. This mutation is located in S4-transmembrane segments of a protein domain II, which is considered the channel voltage sensor segment. The substitution of an arginine by a neutral glutamine determines a change in the 3D structure and in the electrical activity of the P/Q calcium channel, bringing a shift in activation and inactivation dependence to a more negative potential. It is also thought that the inactivation recovery of p.Arg583Gln mutant is slower than normal, leading to accumulation of many inactivated calcium channels during depolarization. The mutation has been already described in about 30 patients with hemiplegic migraine (HM) and ataxia [4–8], who are summarized in Table 1. Phenotype of p.Arg583Gln carriers seems to be quite heterogeneous and not always including cerebellar ataxia. Some isolated and familial carriers have been reported to be asymptomatic or presenting migraine without ataxia [3–8]. This finding is consistent with an incomplete disease penetrance or alternatively with the late disease onset [3, 6], given the young age of most asymptomatic cases. However, despite the heterogeneous phenotypic spectrum, some common characteristics can be drawn. First, cerebellar signs appeared mostly in advanced age and mainly consisted in ataxia and dysmetria with intention tremor, whereas nystagmus was never described [3, 7]. Second, in most cases cerebellar signs are progressive and permanent making the phenotype of p.Arg583Gln mutations more similar to SCA6 than EA2. Lastly, except for a few cases in which migraine was triggered by fever or head trauma, in most cases, a provoking event was not detectable [5, 7]. Our case, except for relatively small attack duration, does not differ from other literature observations. However, the small number of reported patients and the lack of neuroimaging data make it difficult to define a clearer p.Arg583Gln phenotype. Interestingly, acetazolamide seems to be effective in p.Arg583Gln symptomatic carriers [7]. The availability of therapeutic options supports genetic screening for FHM/SHM also in atypical form of hemiplegic migraine with cerebellar symptoms after exclusion of other possible causes.

**Table 1:** CACNA1A R583Q reported mutation in FHM/SHM associated with ataxia in literature

Authors	Type of study	*N* pts	Age of MA onset range/mean	Migraine/headache Features	Frequency	Duration (h)	Cerebellar sign and symptoms	Other clinical features	Age at onset of cerebellar signs	Cerebral MRI/CT
Battistini et al. [7]	Family	3/11	17–40 years	Typical paroxysmal manifestations of HM. 1 pt asymptomatic for HM	12–24/year	Minutes–72 h	Permanent cerebellar ataxia	1 proband with episodes of severe migraine associated with confusion and fever	60 years	Cerebellar atrophy
Ducros et al. [3]	Population	16/117	11.7 ± 8.1 mean ± DS	13/16 HM	N.r.	0.5–125 h	Permanent cerebellar ataxia. In 13/16 pts	Ataxia without nystagmus	N.r.	N.r.
Terwindt et al. [8]	Population	1/27	13 years	Migraine attacks with aphasia, hemiparesis, and confusion	N.r.	<1 h	Permanent cerebellar ataxia	–	N.r.	Normal
Alonso et al. [5]	Family	17 pts from a family	3–23 years	9/17 pts with HM	2/year	24 h	No	Episodes of altered consciousness, focal neurological deficits precipitated or not by minor head trauma	16–50 years	Cerebellar atrophy
Thomsen et al. [6]	Population based	11/291	5–44 years	HM in 8 pts with nausea, vomiting, photofobia and phonofobia. Headache always accompanying attacks	1–8/year	1–48 h	Permanent cerebellar ataxia in 13 pts	Three non affected relatives carried the mutation	N.r.	Cerebellar atrophy in 1 pt
Our case	Case report	1	13 years	HM with transient left hemiparesis, vision loss and paresthesia in right limbs	6/year	<1	Permanent cerebellar ataxia	Decreased level of consciousness, dysarthria, vision loss and sensory deficit alternating in limb side	50 years	Cerebellar atrophy

*N.r*. not reported, *MA* migraine with aura, *FHM/SHM* familial/sporadic hemiplegic migraine, *MRI* magnetic resonance imaging, *CT* computer tomography
